# Supplementary material for: Stabilization of point-defect spin qubits by quantum wells
Source: Nat Commun. 2019 Dec 6;10:5607. doi: 10.1038/s41467-019-13495-6 (PMC6898666; doi:10.1038/s41467-019-13495-6)
Supplement: Supplementary file 1 — Supplementary Information [file 41467_2019_13495_MOESM1_ESM.pdf]

Supplementary Information

for the paper entitled

Stabilization of point-defect spin qubits by quantum wells

V. Ivády,[1,2,*] J. Davidsson,[2] N. Delegan,[3,4] A. L. Falk,[5,6] P. V. Klimov,[5]
S. J. Whiteley,[5] S. O. Hruszkewycz,[4] M. V. Holt,[7] F. J. Heremans,[3,4,5]
N. T. Son,[2] D. D. Awschalom,[3,4,5] I. A. Abrikosov,[2,8] and A. Gali[9,10,†]

[1]*Wigner Research Centre for Physics,*
PO Box 49, H-1525, Budapest, Hungary

[2]*Department of Physics, Chemistry and Biology,*
Linköping University, SE-581 83 Linköping, Sweden

[3]*Center for Molecular Engineering, Argonne National Laboratory, Lemont, IL USA*

[4]*Materials Science Division, Argonne National Laboratory, Lemont, IL USA*

[5]*Pritzker School of Molecular Engineering,*
University of Chicago, Chicago, IL, USA

[6]*IBM T.J. Watson Research Center, Yorktown Heights, NY, USA*

[7]*Center for Nanoscale Materials, Argonne National Laboratory, Lemont, IL, USA*

[8]*Materials Modeling and Development Laboratory,*
National University of Science and Technology 'MISIS', 119049 Moscow, Russia

[9]*Wigner Research Centre for Physics, Hungarian Academy of Sciences,*
PO Box 49, H-1525, Budapest, Hungary

[10]*Department of Atomic Physics, Budapest University of Technology and Economics,*
Budafoki út 8., H-1111 Budapest, Hungary

SUPPLEMENTARY NOTE 1: APPEARANCE OF PL6 CENTERS IN THE NEAR SURFACE REGION

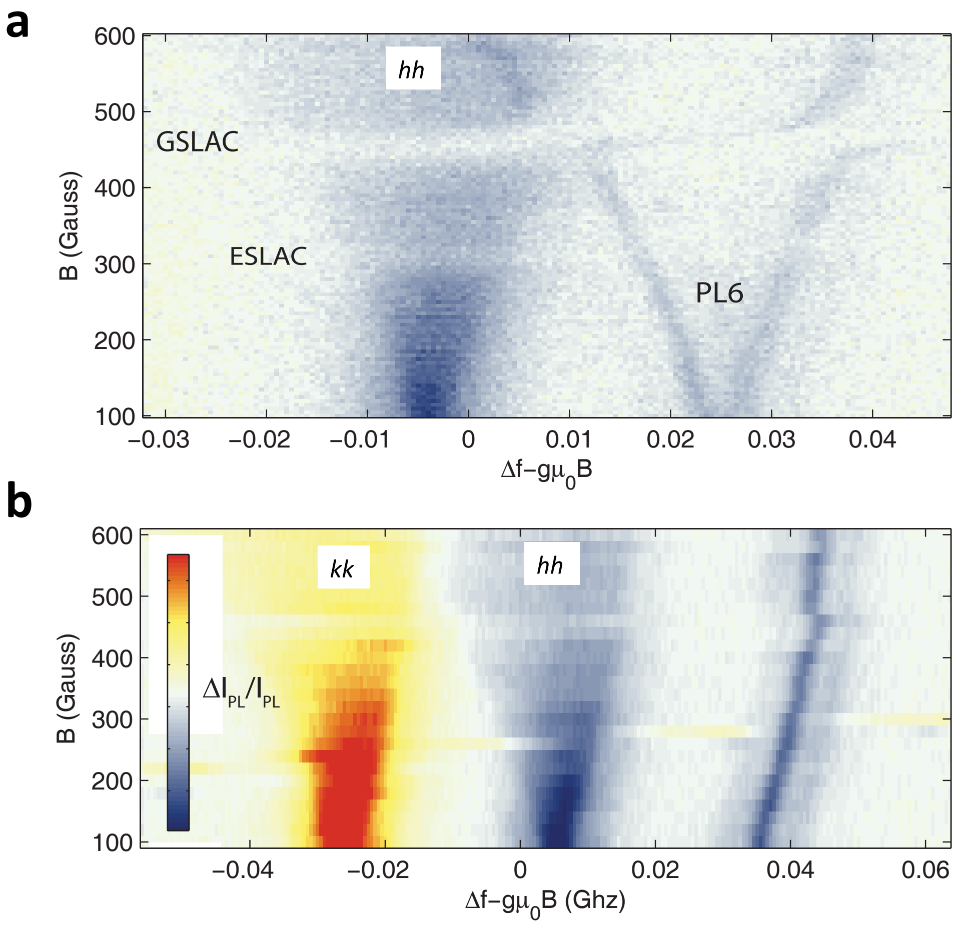

Supplementary Figure 1. PL6 room temperature qubit is a near surface center. **a** Inhomogeneous field Zeeman broadening and splitting of hh divacancy and PL6 qubit ODMR lines, respectively. The splitting of PL6 ODMR line indicates that such centers appears only at the front and back surface of the sample. ESLAC and GSLAC magnetic field regions are labeled that corresponds to excited state and ground state level anticrossing (Supplementary Reference [1]), respectively. In those magnetic field regions the electron spin and neighbor nuclear spins mix that reduces the ODMR contrast (Supplementary Reference [1]). This effect is not relevant in the context. **b** After light chemical etching on one side, the corresponding left branch of PL6 ODMR line disappears. Note that the corresponding resonant frequencies are shifted with respect to **a** because of the smaller thickness of the sample.

In order to facilitate the fabrication of PL6 qubits, we carry out optically detected magnetic resonance (ODMR) experiments to locate PL6 centers in 4H-SiC. We apply inhomogeneous magnetic field along the c-axis of an as-grown 4H-SiC sample and record the ODMR signal of bulk divacancy configurations and PL6 qubit, see Supplementary Figure 1a. hh and kk divacancies are continuously distributed in the sample, thus the varying Zeeman splitting of the centers causes a blurred ODMR signal for these configurations. On the other hand, the signal of PL6 center exhibits two sharp lines corresponds to the maximal and minimal magnetic fields observable at the surfaces of the sample. These results indicate that PL6 centers appear only at the front and back surface of the sample. Furthermore, by etching off one of the surfaces by using an SF_6 and Ar/Cl_2 inductively coupled plasma (ICP) process, etching depth was ≈ 2 μm, the corresponding PL6 ODMR line disappears, see Supplementary Figure 1b. This result indicates that PL6 qubits do not appear directly because of the influence of the surface, but most likely because of the presence of other defects in the near-surface region, e.g. the stacking faults. Indeed, PL6 centers do not show up on the etched side, thus the source of this signal was completely removed. As we mentioned in the main text, the expected higher stacking fault concentration at the surface implies the appearance of new divacancy configurations in this region, which coincides with the expectations of our model for PL6 qubit.

SUPPLEMENTARY NOTE 2: PROPERTIES OF DIVACANCY POINT DEFECTS, ZERO-FIELD-SPLITTING

In Supplementary Table 1 and Supplementary Table 2 we provide theoretical and experimental zero-field-splitting parameters for axial and basal plane divacancy configurations considered in the study, see Fig. 2D in the main text. For details on the calculation see the main text.

SUPPLEMENTARY NOTE 3: PROPERTIES OF DIVACANCY POINT DEFECTS, HYPERFINE SPLITTING

In Supplementary Table 3 and Supplementary Table 4 we provide theoretical and experimental hyperfine splitting values for first neighbor ^{13}C and second neighbor ^{29}Si nuclei sites of axial and basal plane divacancies, respectively. Spin density of an axial divacancy and the considered neighboring sites are depicted in Fig. 3A in the main text. Details on the hyperfine calculations

Supplementary Table 1. Theoretical and experimental zero-field-splitting values (D) for axial divacancy configurations.

Model		Exp.	
Conf.	D [MHz]	Center	D [MHz]
hh-4H	1414	PL1	1336
h_1h_1-ssf	1416		
h_2h_2-ssf	1415		
kk-4H	1374	PL2	1305
k_1k_1-ssf	1376		
k_3k_3-ssf	1369		
k_2k_2-ssf	1454	PL6	1365

Supplementary Table 2. Theoretical and experimental zero-field-splitting parameters (D and E) for basal divacancy configurations.

Model			Exp.		
Conf.	D [MHz]	E [MHz]	Center	D [MHz]	E [MHz]
kh-4H	1322	71	PL3	1222	82
k_3h_2-ssf	1315	65			
hk-4H	1374	19	PL4	1334	19
hk_2-ssf	1366	24			
k_1h-ssf	1336	19			
k_2k_1-ssf	1357	140			

can be found in the main text.

Supplementary Table 3. Calculated and measured hyperfine splitting (A_z) for the axial divacancy configurations. The considered first and second neighbor sites are shown in Fig. 3A in the main text. The experimental results were reported in Supplementary Reference [1]. All values are provided in MHz unit.

Model				Exp.		
Conf.	$\mathrm{C_I}$	$\mathrm{Si_{IIa}}$	$\mathrm{Si_{IIb}}$	Center	$\mathrm{Si_{IIa}}$	$\mathrm{Si_{IIb}}$
hh-4H	58.2	11.7	8.6	PL1	12.3	9.2
h_1h_1-ssf	58.4	11.7	8.6			
h_2h_2-ssf	58.2	11.7	8.7			
kk-4H	53.6	12.6	9.6	PL2	13.2	10.0
k_1k_1-ssf	53.8	12.6	9.6			
k_3k_3-ssf	53.5	12.7	9.6			
k_2k_2-ssf	60.3	11.7	8.8	PL6	12.5	9.6

Supplementary Table 4. Calculated hyperfine splitting (A_z) for basal plane divacancy configurations. The considered first and second neighbor sites are shown in Fig. 3A in the main text. Due to the low symmetry of basal plane divacancy configurations we distinguish symmetrically non-equivalent sites in the neighbor shells. All values are provided in MHz unit.

Conf.	$\mathrm{C_{I\text{-}1}}$	$\mathrm{C_{I\text{-}2}}$	$\mathrm{Si_{IIa\text{-}1}}$	$\mathrm{Si_{IIa\text{-}2}}$	$\mathrm{Si_{IIb\text{-}1}}$	$\mathrm{Si_{IIb\text{-}2}}$	$\mathrm{Si_{IIb\text{-}3}}$
kh-4H	117.8	56.4	11.5	12.5	9.6	9.7	9.8
k_3h_2-ssf	118.0	56.2	11.6	12.4	9.6	9.7	9.9
hk-4H	121.0	60.7	11.1	11.5	8.2	9.2	11.9
hk_2-ssf	120.8	60.5	11.1	11.6	8.2	9.3	11.9
k_1h-ssf	120.0	56.6	12.0	12.3	9.3	9.4	10.2
k_2k_1-ssf	116.0	63.8	11.0	12.1	9.0	9.0	9.2

Supplementary Table 5. Theoretical ZPL energies for all the considered axial divacancy configurations, see Fig. 2D in the main text, as compared with the available experimental data[2].

Model		Exp.	
Conf.	ZPL [eV]	Center	ZPL [eV]
hh-4H	0.92	PL1	1.095
h_1h_1-ssf	0.92		
h_2h_2-ssf	0.92		
kk-4H	0.94	PL2	1.096
k_1k_1-ssf	0.93		
k_3k_3-ssf	0.94		
k_2k_2-ssf	0.97	PL6	1.194

SUPPLEMENTARY NOTE 4: PROPERTIES OF DIVACANCY POINT DEFECTS, ZERO-PHONON PHOTO LUMINESCENCE LINE

The energy of the lowest energy optically excited state is obtain via constrain occupation DFT method in PBE[3] functional calculations in a 2816 atom supercell with $2 \times 2 \times 1$ k-point set. Note that such calculations satisfy the numerical convergence criteria required for reproducing the order of the zero-phonon photo luminescence (ZPL) energies of different divacancy configurations[4], which is the focus in this section. We also note that the excited states of the high symmetry c-axis-oriented divacancies are dynamic Jahn-Teller (DJT) unstable. We approximated the DJT excited state energies by the static Jahn-Teller excited state energies in these cases. As the energy of the Jahn-Teller distortion is $\approx$30 meV, neglect of electron phonon coupling presumable causes uncertainties in the ZPL energy differences in the order of 10 meV.

The calculated and experimental ZPL energies of the axial divacancies are provided in Supplementary Table 5. Considering the small splitting of the ZPL lines and the estimated error bar of the theoretical results, only limited statements can be made. Similar classes of the ZPL energies can be observed as for the hyperfine and the zero-field-splitting parameters seen in the main text, however, the splitting between the PL1 and PL2 configurations are highly overestimated. Most importantly, the ZPL energy of k_2k_2-ssf configuration is well separated from the ZPL energy of other axial configurations and possesses the largest ZPL energy. These observations further support the

identification of PL6 qubit as k_2k_2-ssf configuration.

The ZPL energies of the basal plane oriented configurations closely follow each other and several configurations have been found closer to each other than 10 meV, which is our numerical accuracy in these calculations. Consequently, no conclusive statement can be made from the ZPL results of the basal plane configurations.

SUPPLEMENTARY NOTE 5: PROPERTIES OF DIVACANCY POINT DEFECTS, SINGLE PARTICLE OPTICAL ABSORPTION SPECTRUM

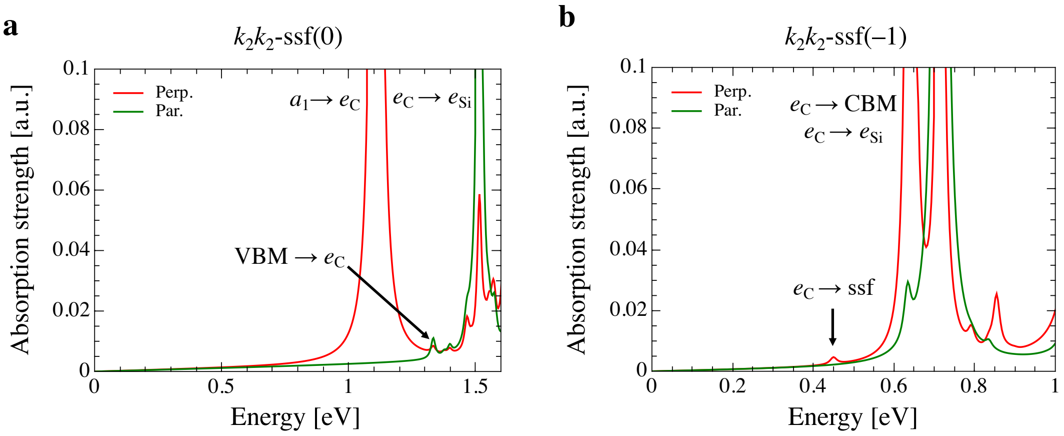

Supplementary Figure 2. Single particle absorption spectrum of **a** neutral and **b** negatively charged k_2k_2-ssf (PL6) divacancy configuration. Intra defect transitions and transitions between the valence band maximum (VBM), conduction band minimum (CDM), and single stacking faults (ssf) states and the divacancy e_C state are labeled.

The size of the stacking fault model, 1536 atom in this case, allows us to investigate the absorption spectrum only on single particle level by using the semilocal PBE[3] functional. Due to the band gap underestimation error of semilocal DFT functionals, the transition energies between delocalized host and localized defect states are substantially underestimated. On the other hand, the relative position of the absorption peaks and the transition strength might be obtained with reasonable accuracy even within this approximation.

The single particle absorption spectrum can be seen in Supplementary Figure 2a and b for the neutral and negative charge state of k_2k_2-ssf divacancy configuration, respectively. In the neutral case, the lowest energy non-intra-defect transition happens between the VBM and e_C defect state.

This result supports the negative charge state to be identified as the dark state of divacancy defect. Comparing the single particle absorption spectra with higher level theory absorbtion spectra reported in Supplementary Reference [5], one can see that this statement is further confirmed. On the other hand, the transition energy of $e_{\mathrm{C}} \rightarrow e_{\mathrm{Si}}$ intra-defect transition falls below the VBM $\rightarrow e_{\mathrm{C}}$ transition in Supplementary Reference [5], which is in contrast to the single particle spectrum depicted in Supplementary Figure 2. The differences can be attributed to the electron-hole interaction that might be enhanced for localized defect states. Note that similar effect can be expected in the case of negative charge state, i.e. the $e_{\mathrm{C}} \rightarrow e_{\mathrm{Si}}$ transition may have lower energy than the $e_{\mathrm{C}} \rightarrow$ CBM and $e_{\mathrm{C}} \rightarrow$ ssf transitions. Note, on the other hand, that intra-defect transitions cannot change the charge state of the defect thus the defect remains in its dark negative charge state. Therefore, the actual position of $e_{\mathrm{C}} \rightarrow e_{\mathrm{Si}}$ transition does not influence our statements made for non-intra-defect transitions.

As can be seen in Supplementary Figure 2b, the presence of the divacancy defect states do not alter the appearance of the single stacking fault states below the CBM. Most importantly, there is a finite transition strength for $e_{\mathrm{C}} \rightarrow$ ssf transitions that may thus allow for bright state re-pumping processes.

Due to the overlap with the strong intra-defect $a_1 \rightarrow e_{\mathrm{C}}$ and $e_{\mathrm{C}} \rightarrow e_{\mathrm{Si}}$ transitions, relative transition strength of free-to-bound and bound-to-free like transitions cannot be reliably determined from the absorption spectra. Therefore, in the following section we provide transition dipole moments for pairs of Kohn-Sham defect states.

---

* ivady.viktor@wigner.mta.hu

† gali.adam@wigner.mta.hu

[1] A. L. Falk, P. V. Klimov, V. Ivády, K. Szász, D. J. Christle, W. F. Koehl, A. Gali, and D. D. Awschalom, Phys. Rev. Lett. **114**, 247603 (2015).

[2] A. L. Falk, B. B. Buckley, G. Calusine, W. F. Koehl, V. V. Dobrovitski, A. Politi, C. A. Zorman, P. X.-L. Feng, and D. D. Awschalom, Nature Commun. **4**, 1819 (2013).

[3] J. P. Perdew, K. Burke, and M. Ernzerhof, Phys. Rev. Lett. **77**, 3865 (1996).

[4] J. Davidsson, V. Ivady, R. Armiento, N. T. Son, A. Gali, and I. A. Abrikosov, New Journal of Physics **20**, 023035 (2018).

[5] M. Bockstedte, F. Schütz, T. Garratt, V. Ivády, and A. Gali, npj Quantum Materials **3**, 31 (2018).
